# Association of ratios of visceral fat area/subcutaneous fat area and muscle area/standard body weight at T12 CT level with the prognosis of acute respiratory distress syndrome

**DOI:** 10.1016/j.pccm.2024.05.004

**Published:** 2024-06-20

**Authors:** Hui Shen, Ying He, Fan Lu, Xiaoting Lu, Bining Yang, Yi Liu, Qiang Guo

**Affiliations:** a Department of Emergency, The Fourth Affiliated Hospital of Soochow University (Suzhou Dushu Lake Hospital), Suzhou, Jiangsu 215000, China; b Department of Pulmonary and Critical Care Medicine, The Fourth Affiliated Hospital of Soochow University (Suzhou Dushu Lake Hospital), Suzhou, Jiangsu 215000, China; c Institute of Critical Care Medicine, Soochow University, Suzhou, Jiangsu 215000, China; d Medical Center of Soochow University, Suzhou, Jiangsu 215000, China; e Department of Emergency and Critical Care Medicine, The First Affiliated Hospital of Soochow University, Suzhou, Jiangsu 215000, China

**Keywords:** Acute respiratory distress syndrome, Prognosis, Visceral fat area/subcutaneous fat area, Muscle area/standard body weight

## Abstract

**Background:**

It is well-known that body composition metrics can influence the prognosis of various diseases. This study investigated how body composition metrics predict acute respiratory distress syndrome (ARDS) prognosis, focusing on the ratio of visceral fat area (VFA) to subcutaneous fat area (SFA), SFA to standard body weight (SBW), VFA to SBW, and muscle area (MA) to SBW. These metrics were assessed at the level of the twelfth thoracic vertebra (T12 computed tomography [CT] level) to determine their correlation with the outcomes of ARDS. The goal was to utilize these findings to refine and personalize treatment strategies for ARDS.

**Methods:**

Patients with ARDS admitted to the intensive care units (ICUs) of three hospitals from January 2016 to July 2023 were enrolled in this study. Within 24 hours of ARDS onset, we obtained chest CT scans to measure subcutaneous fat, visceral fat, and muscle area at the T12 level. We then compared these ratios between survivors and non-survivors. Logistic regression was employed to identify prognostic risk factors. Receiver operating characteristic (ROC) curve analysis was utilized to determine the optimal cutoff for predictors of in-hospital mortality. Based on this cutoff, patients with ARDS were stratified. To reduce confounding factors, 1:1 propensity score matching (PSM) was applied. We conducted analyses of clinical feature and prognostic differences pre- and post-PSM between the stratified groups. Additionally, Kaplan–Meier survival curves were generated to compare the survival outcomes of these groups.

**Results:**

Of 258 patients with ARDS, 150 survived and 108 did not. Non-survivors had a higher VFA/SFA ratio (*P* <0.001) and lower SFA/SBW and MA/SBW ratios (both *P* <0.001). Key risk factors were high VFA/SFA ratio (OR=2.081; *P*=0.008), age, acute physiology and chronic health evaluation (APACHE) II score, and lactate levels, while MA/SBW and albumin were protective. Patients with a VFA/SFA ratio ≥0.73 were associated with increased mortality, while those with an MA/SBW ratio >1.55 cm²/kg had lower mortality, both pre- and post-PSM (*P*=0.001 and *P* <0.001, respectively). Among 170 patients with pulmonary-origin ARDS, 87 survived and 83 did not. The non-survivor group showed a higher VFA/SFA ratio (*P* <0.001) and lower SFA/SBW and MA/SBW (*P*=0.003, *P* <0.001, respectively). Similar risk and protective factors were observed in this cohort. For VFA/SFA, a value above the cutoff of 1.01 predicted higher mortality, while an MA/SBW value below the cutoff of 1.48 cm²/kg was associated with increased mortality (both *P* <0.001 pre-/post-PSM).

**Conclusions:**

Among all patients with ARDS, the VFA to SFA ratio, MA to SBW ratio at the T12 level, age, APACHE II score, and lactate levels emerged as independent risk factors for mortality.

## Introduction

Acute respiratory distress syndrome (ARDS) is a severe lung disorder caused by inflammation, leading to increased pulmonary vessel permeability, bilateral lung edema, and persistent hypoxemia. ARDS arises from intrapulmonary or extrapulmonary causes, characterized by either direct lung damage or a systemic inflammatory response.^1^^,^^2^ Its etiology might be infectious or noninfectious, causing localized inflammation with direct pulmonary damage or a broader systemic inflammatory response.^3^ Despite advances in understanding and treatment, ARDS mortality rates remain high, with mild, moderate, and severe cases showing 35%, 45%, and 46% mortality rates, respectively.^4^^,^^5^ Evaluating prognostic indicators is crucial for clinicians to anticipate outcomes, identify risk factors, and make informed treatment decisions.

Obesity, typically a mortality risk in cardiovascular diseases and diabetes, shows a paradoxical decrease in mortality in ARDS, known as the “obesity paradox”.^6^^,^^7^ Its protective role in ARDS is not fully understood, but it is hypothesized that adipose tissue might mitigate inflammation through immunomodulating agents like lipocalin and interleukin-10 (IL-10), thereby improving survival.^8^^,^^9^ Body mass index (BMI), commonly used to assess obesity, is limited in its application to ARDS because of its failure to consider edema, fluid shifts, and specific fat distributions. Prior studies have used abdominal CT scans to measure subcutaneous and visceral fat, providing a more refined obesity assessment in relation to ARDS.^10^^,^^11^ Our research focuses on the relationship between chest CT-quantified subcutaneous and visceral fat areas and ARDS outcomes. Chest CT scans, more accessible and convenient during hospitalization, are preferred over abdominal scans. Additionally, reduced skeletal muscle, as measured by CT-derived muscle area (MA), is associated with ARDS onset and increased mortality post-pulmonary resection.^12^^,^^13^

Body composition evaluations at the third lumbar vertebra (L3) are established indicators of body fat and muscle mass.^14^ However, abdominal CT scans, which typically exclude L3, are not standard in ARDS diagnosis, unlike chest CT scans. Recent studies indicate that measurements of fat and MA at the T12 level are comparable to those at L3.^15^^,^^16^ Consequently, this study utilizes T12-level CT scans, supported by relevant literature, to examine the association between body composition and ARDS prognosis. We quantified the subcutaneous fat area (SFA), visceral fat area (VFA), and MA at T12 using patients’ chest CT scans. Additionally, we calculated the VFA/SFA ratio to explore the relationship between chest fat distribution and ARDS prognosis. Considering the influence of height on chest dimensions, using CT-derived fat and muscle measurements alone for obesity assessment may be insufficient. Therefore, we adjusted these metrics based on the patient's standard body weight (SBW) for a more accurate analysis of the links between chest fat, muscle, and ARDS outcomes.

## Methods

### Ethical approval

All procedures were conducted in strict accordance with the ethical standards set forth by the institutional and national research committees, as well as the 2013 revision of the *Declaration of Helsinki*. The study protocol received approval from the Ethics Committee of The Fourth Affiliated Hospital of Soochow University (Suzhou Dushu Lake Hospital) (Approval No. 231027). A waiver of written informed consent was granted by the institutional review board, considering the retrospective nature of the study and the use of anonymized patient data.

### Study population and observation indicators

In this retrospective clinical study, we included patients with ARDS treated in the intensive care units (ICUs) of the First Affiliated Hospital of Soochow University, the Second Affiliated Hospital of Soochow University, and the Second People's Hospital of Kunshan from January 2016 to July 2023. The primary observational indicators included the patient's age, sex, height, weight, smoking history, underlying medical conditions, and etiology of ARDS. Upon ICU admission, additional clinical parameters were recorded, including neutrophil count, albumin (ALB) level, hemoglobin (Hb) level, platelet (PLT) count, aspartate aminotransferase (AST) level, alanine aminotransferase (ALT) level, lactate (Lac) level, and oxygenation index (OI). Furthermore, we documented the Acute Physiology and Chronic Health Evaluation (APACHE) II and Sequential Organ Failure Assessment (SOFA) scores within 24 hours of ICU admission. Chest CT scans were conducted to evaluate the transverse-to-anteroposterior diameter ratio of the chest wall at the T12 level, 24 hours post-ARDS onset. Additionally, measurements were taken for SFA, VFA, and MA. To establish relevant indicators, we calculated the ratios of VFA/SFA, SFA/SBW, VFA/SBW, and MA/SBW. The study further recorded the duration of mechanical ventilation, duration of ICU stay, and in-hospital mortality.

### Inclusion and exclusion criteria

Inclusion criteria: (1) adult (age ≥18 years), (2) diagnosed with ARDS according to the Berlin criteria after admission, (3) undergoing chest CT examination within 24 hours of ARDS onset, and (4) admitted to the ICU for treatment within 24 hours after ARDS diagnosis.

Exclusion criteria: (1) patients who died within 24 hours of hospital admission or ICU admission, (2) patients with mechanical ventilation for less than 48 hours, (3) patients whose diaphragm was not scanned at the T12 level because of severe emphysema, large pleural effusion, or other reasons, (4) patients with poor-quality or artifact-affected chest CT scans or scans that did not reach the T12 level, (5) patients who were pregnant or had a tumor diagnosis, (6) patients with incomplete clinical data that could not be included in the statistical analysis.

### CT data collection

**Fig. 1:**
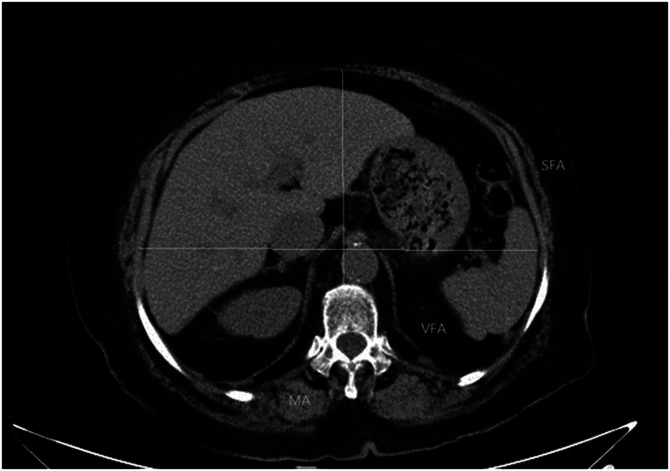
CT at the level of the twelfth thoracic vertebra. MA: Muscle area; SFA: Subcutaneous fat area; VFA: Visceral fat area.

In this study, chest imaging was performed using a dual-source spiral CT scanner (Siemens Shanghai Medical Equipment Ltd, Shanghai, China), set to a slice thickness and interval of 5 mm. The acquired CT images were then processed using picture archiving and communication system (PACS) software. On these scans, muscle tissues exhibited CT values ranging from −29 to 150 Hounsfield units (HU), while fat tissues were characterized by values between −190 and −30 HU. We conducted quantitative analyses of SFA, VFA, MA, transverse thoracic diameter, and anterior–posterior thoracic diameter at the T12 level. This analysis utilized PACS software, leveraging the specific CT values for fat and muscle tissues (see Fig. 1 for detailed imaging). Furthermore, the study calculated the VFA/SFA ratio and normalized the cross-sectional areas of both fat and muscle tissues to the SBW. This normalization enabled the calculation of ratios such as SFA/SBW, VFA/SBW, and MA/SBW, providing key indicators for our research.

### Definitions and grading criteria

#### Definition of ARDS and classification of ARDS etiology

The definition of ARDS refers to the Berlin Diagnostic Criteria for ARDS.^17^ The categorization of ARDS is delineated by its origin, distinguishing between pulmonary and extrapulmonary types. Pulmonary ARDS develops from conditions that directly compromise lung function. These conditions include severe infections in the lungs, inhalation of toxic gases, and trauma resulting in lung tissue bruising. In contrast, extrapulmonary ARDS arises from systemic issues that affect the body more broadly, beyond the lungs. Key causes of this type include sepsis, pancreatitis, and traumas unrelated to the chest area.

#### BMI, obesity, and SW

The BMI was calculated from a person's height-to-weight ratio squared. It is categorized as: underweight (BMI <18.5 kg/m^2^), normal (18.5 kg/m^2^ ≤ BMI <25 kg/m^2^), overweight (25 kg/m^2^ ≤ BMI <30 kg/m^2^), obese (30 kg/m^2^ ≤ BMI <40 kg/m^2^), and morbidly obese (BMI ≥40 kg/m^2^). For men's standard weight: standard weight (kg) = (height [cm]−80) × 70%. For women: standard weight (kg) = (height [cm]−70) × 60%.^18^

#### Propensity score matching (PSM)

Observational studies may reflect differences in baseline attributes rather than the variables under observation, leading to potential biases. Therefore, many studies use PSM to counteract multiple confounders.^19^^,^^20^ PSM pairs individuals based on similar or closely matched propensity scores, reducing selection bias and other confounding influences on results.^21^ In this study, we matched participants based on factors such as age, sex, APACHE II score, albumin level, and other relevant variables to ensure comparability between groups.

#### Disease-scoring criteria

The APACHE II score, accounting for acute physiological data, age, and chronic health issues, is the peak score within the first 24 hours of admission, with higher scores indicating increased risk of mortality.^22^ The SOFA score, comprising six organ-function criteria rated between 0 and 4, reflects that rising scores denote deteriorating prognoses.^23^

### Research steps

Patients with ARDS were divided into survival and death groups based on their prognosis. We compared basic clinical data, laboratory parameters, and the ratios of VFA/SFA, SFA/SBW, VFA/SBW, and MA/SBW at the T12 level between these groups. Using logistic regression, we identified ARDS mortality risk factors, selecting variables with *P*-value <0.1 for multiple regression analysis to ascertain independent risk factors. Receiver operating characteristic (ROC) curves were generated for the ARDS mortality cohort to determine optimal cut-off values, evaluating the sensitivity and specificity of predictive indicators. Patients with ARDS were then classified into low (variable <cut-off) and high (variable ≥cut-off) risk groups based on these cut-off values. We compared their clinical characteristics and prognostic differences. To mitigate baseline variations, we implemented PSM at a 1:1 ratio using a 0.02 caliper. We examined clinical data, laboratory markers, and key prognostic indicators before and after matching, supplementing our analysis with Kaplan–Meier survival plots. Finally, we explored the association between these indicators and the prognosis of pulmonary ARDS.

### Statistical analysis

Data were analyzed using IBM SPSS Statistics for Windows, version 25.0 (IBM Corp., Armonk, NY, USA) with a significance threshold of *P* <0.05. Before PSM, continuous variables were evaluated using independent *t*-test or Mann–Whitney *U* test, depending on the distribution. They were presented as mean ± standard deviation or median (Q_1_, Q_3_). Categorical data, displayed as percentages and counts, were tested with chi-squared or Fisher's exact tests. After matching, continuous variables underwent paired *t*-tests or Mann-Whitney *U* tests, with results similarly displayed. Categorical variables, presented as counts and percentages, were evaluated using McNemar's test. Logistic regression, including univariable and multivariable analyses, identified ARDS prognosis risk factors. For ARDS mortality prediction, ROC curve analysis determined optimal values, sensitivity, and specificity. Kaplan–Meier curves were generated using GraphPad Prism, version 8 for Windows (GraphPad Software, San Diego, CA, USA).

## Results

### Relationship between adipose areas and MA at the T12 CT level and ARDS prognosis

#### Comparison of clinical features in patients with ARDS grouped by outcome

A total of 258 patients with ARDS met the inclusion criteria and participated in this study. As detailed in Table 1, patients were divided into two groups based on outcomes: survivors (*n*=150) and non-survivors (*n*=108). We undertook a comparative analysis of their clinical characteristics to identify prognostic factors. Our findings indicated that non-survivors were significantly older than survivors (*P* <0.001). Moreover, non-survivors had a reduced BMI (*P*=0.031) and a higher prevalence of severe ARDS (29.6% *vs*. 18.0%, *P*=0.039). Upon ICU admission, non-survivors showed significantly higher APACHE II scores and lactate levels (both *P* <0.001), whereas their albumin levels were significantly lower (*P*=0.019). In terms of thoracic adipose and muscular measurements, non-survivors consistently displayed significantly lower values for SFA, SFA/SBW, MA, and MA/SBW (all *P* <0.001). Additionally, the ratios of VFA/SFA and thoracic transverse diameter to anteroposterior diameter were notably increased in the non-survivors (*P* <0.001 and *P*=0.011, respectively). Yet, the ratio of VFA/SBW, although elevated in the death group, was not statistically significant (*P*=0.810). There were no significant differences between the two groups in terms of sex, smoking history, underlying diseases, SOFA scores, other lab metrics, or the oxygenation index (PaO_2_/FiO_2_).

**Table 1:** Comparison of clinical features in patients with ARDS grouped by outcome.

Variables	Survival group (*n*=150)	Death group (*n*=108)	Statistics	*P* values
Age (years)	65.00 (48.00, 76.00)	75.00 (64.50, 82.00)	*U* = 5460.5	<0.001
Male	107 (71.3)	73 (67.6)	χ² = 0.414	0.519
BMI (kg/m²)	23.05 (20.81, 26.18)	22.23 (20.22, 24.57)	*U* = 7032.0	0.031
Smoking history	45 (30.0)	26 (24.1)	χ² = 1.105	0.293
Underlying diseases				
Hypertension	77 (51.3)	68 (63.0)	χ² = 3.470	0.063
Diabetes	34 (22.7)	28 (25.9)	χ² = 0.364	0.546
Coronary heart disease	7 (4.7)	8 (7.4)	χ² = 0.861	0.353
Hyperlipidemia	7 (4.7)	3 (2.8)	-	0.528
COPD	12 (8.0)	9 (8.3)	χ² = 0.007	0.923
Causes of ARDS				
Pulmonary infection (bacteria, virus, etc.)	80 (53.3)	82 (75.9)	χ² = 12.381	<0.001
Aspiration of gastric contents	4 (2.7)	0 (0)	-	1.000
Inhalation of irritant gases	1 (0.7)	0 (0)	-	1.000
Drowning	2 (1.3)	1 (0.9)	-	1.000
Abdominal infection leading to sepsis	4 (2.7)	4 (3.7)	-	0.725
Urinary system infection leading to sepsis	4 (2.7)	2 (1.9)	-	0.683
Pancreatitis	25 (16.7)	5 (4.6)	χ² = 9.802	0.002
Trauma (outside the thorax)	12 (8.0)	2 (1.9)	-	0.050
Others	18 (12.0)	12 (11.1)	χ² = 0.037	0.848
ARDS classification on ICU admission			χ² = 6.500	0.039
Mild	42 (28.0)	33 (30.6)		
Moderate	81 (54.0)	43 (39.8)		
Severe	27 (18.0)	32 (29.6)		
APACHE II score	13.00 (10.00, 16.00)	16.00 (13.00, 20.00)	*U* = 5870.0	<0.001
SOFA score	7.00 (5.00, 9.00)	8.00 (5.00, 10.00)	*U* = 7740.0	0.108
Laboratory indicators on ICU admission				
Neutrophil count (× 10⁹/L)	9.41 (6.00, 13.86)	9.51 (5.98, 14.65)	*U* = 8050.0	0.835
Platelet count (× 10⁹/L)	168.50 (104.75, 226.00)	149.00 (75.25, 226.75)	*U* = 7300.0	0.080
Hemoglobin (g/L)	115.34±28.06	111.64±27.32	*t* = 1.055	0.292
Albumin (g/L)	30.88±6.40	29.05±5.78	*t* = 2.369	0.019
AST (U/L)	40.15 (23.80, 66.98)	37.00 (24.25, 99.50)	*U* = 7580.0	0.489
ALT (U/L)	27.45 (15.60, 60.18)	27.65 (18.05, 60.35)	*U* = 7605.0	0.535
Arterial blood gas on ICU admission				
PaO₂/FiO₂ (mmHg)	172.65 (121.01, 227.48)	168.78 (91.67, 246.67)	*U* = 7090.0	0.403
Lactate (mmol/L)	1.60 (1.10, 2.80)	2.65 (1.80, 5.28)	*U* = 5655.0	<0.001
Thoracic fat and muscle area				
SFA (cm²)	73.15 (48.24, 107.63)	51.73 (27.23, 88.24)	*U* = 5950.0	<0.001
VFA (cm²)	60.78 (33.58, 97.12)	62.48 (29.14, 103.48)	*U* = 8200.0	0.969
MA (cm²)	91.33 (72.88, 111.35)	77.99 (60.90, 84.45)	*U* = 6635.0	<0.001
Thoracic fat and muscle area adjusted for standard weight				
SFA/SBW (cm²/kg)	1.21 (0.81, 1.87)	0.88 (0.46, 1.41)	*U* = 6005.0	<0.001
VFA/SBW (cm²/kg)	1.03 (0.61, 1.60)	1.08 (0.56, 1.72)	*U* = 8190.0	0.810
MA/SBW (cm²/kg)	1.49 (1.21, 1.80)	1.28 (1.06, 1.45)	*U* = 6750.0	<0.001
VFA/SFA	0.76 (0.50, 1.30)	1.06 (0.68, 1.71)	*U* = 6055.0	<0.001
Thorax width/depth	2.04 (1.84, 2.30)	2.18 (1.97, 2.35)	*U* = 7000.0	0.011

Data are presented as mean±standard deviation, median (Q_1_, Q_3_) or *n* (%). ALT: Alanine aminotransferase; APACHE: Acute Physiology and Chronic Health Evaluation; ARDS: Acute respiratory distress syndrome; AST: Aspartate aminotransferase; BMI: Body mass index; COPD: Chronic obstructive pulmonary disease; FiO_2_: Fraction of inspired oxygen; ICU: Intensive care unit; MA: Muscle area; PaO_2_: Partial pressure of oxygen in arterial blood; SBW: Standard body weight; SFA: Subcutaneous fat area; SOFA: Sequential Organ Failure Assessment; VFA: Visceral fat area; -: Not available.

#### Univariable and multivariable logistic regression analysis for mortality in patients with ARDS

As detailed in Table 2, potential prognostic variables for ARDS were first discerned through univariable logistic regression analyses. Factors with a *P*-value below 0.1 were then included in a multivariable logistic regression model. From this refined analysis, several factors stood out as significant. Age was identified to be significantly associated with ARDS mortality, with an odds ratio (OR) of 1.027 (95% confidence interval [CI], 1.003–1.051, *P*=0.030). APACHE II score was also significantly associated with mortality (OR=1.146, 95% CI 1.064–1.234, *P* <0.001). Elevated lactate levels also correlated with increased ARDS mortality (OR=1.116, 95% CI 1.014–1.228, *P*=0.024). The ratio of VFA/SFA was associated with higher ARDS mortality (OR=2.081, 95% CI 1.215–3.563, *P*=0.008). However, two factors surfaced as protective against ARDS mortality. Higher albumin levels were associated with reduced mortality (OR=0.937, 95% CI 0.889–0.988, *P*=0.015). An increased ratio of muscle area to SBW was significantly protective (OR=0.060, 95% CI 0.018–0.207, *P* <0.001).

**Table 2:** Univariable and multivariable logistic regression analysis for mortality in patients with ARDS.

Variables	Univariable regression	*P* values	Multivariable regression	*P* values
	OR (95% CI)		OR (95% CI)	
Age (years)	1.039 (1.022–1.056)	<0.001	1.027 (1.003–1.051)	0.030
BMI (kg/m^2^)	0.925 (0.868–0.987)	0.018	0.940 (0.875–1.010)	0.097
Hypertension	1.612 (0.973–2.671)	0.064	1.320 (0.800–2.178)	0.275
APACHE Ⅱ score	1.162 (1.098–1.229)	<0.001	1.146 (1.064–1.234)	<0.001
SOFA score	1.067 (0.994–1.146)	0.073	1.055 (0.981–1.136)	0.147
Albumin (g/L)	0.949 (0.910–0.989)	0.014	0.937 (0.889–0.988)	0.015
Lactate (mmol/L)	1.170 (1.065–1.285)	0.001	1.116 (1.014–1.228)	0.024
SFA/SBW (cm^2^/kg)	0.534 (0.374–0.763)	0.001	0.600 (0.400–0.900)	0.015
VFA/SFA	2.061 (1.415–3.001)	<0.001	2.081 (1.215–3.563)	0.008
MA/SBW (cm^2^/kg)	0.157 (0.074–0.332)	<0.001	0.060 (0.018–0.207)	<0.001

APACHE: Acute Physiology and Chronic Health Evaluation; ARDS: Acute respiratory distress syndrome; BMI: Body mass index; CI: Confidence interval; MA: Muscle area; OR: Odds ratio; SBW: Standard body weight; SFA: Subcutaneous fat area; SOFA: Sequential Organ Failure Assessment; VFA: Visceral fat area.

#### ROC curve analysis for ARDS prognosis and analysis of thresholds, sensitivity, and specificity for prognostic factors

**Fig. 2:**
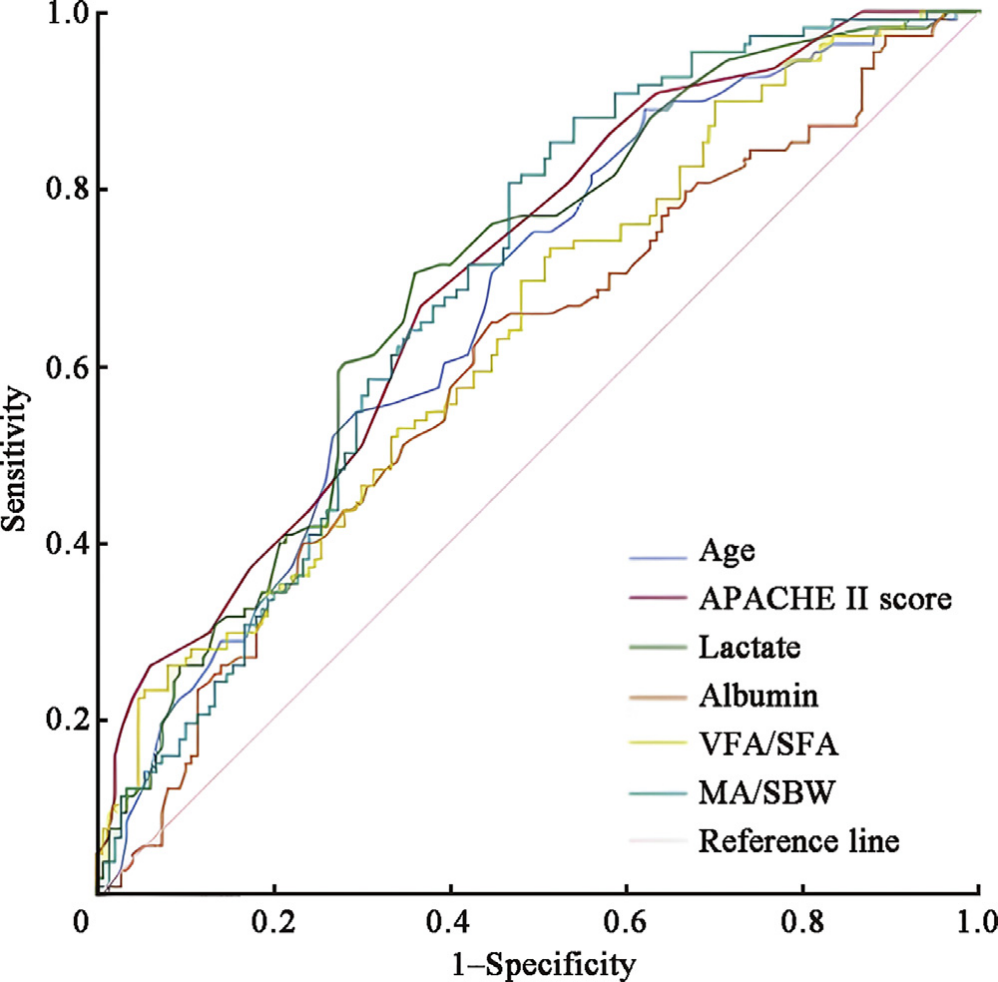
ROC curve for predicting ARDS mortality. APACHE: Acute Physiology and Chronic Health Evaluation; ARDS: Acute respiratory distress syndrome; MA: Muscle area; ROC: Receiver operating characteristic; SBW: Standard body weight; SFA: subcutaneous fat area; VFA: Visceral fat area.

As depicted in Fig. 2 and outlined in Table 3, the ROC curve was employed to ascertain the optimal thresholds for the predictors of ARDS mortality.

**Table 3:** Analysis of thresholds for predictors related to ARDS mortality.

Variables	AUC	95% CI	*P* values	Optimal thresholds	Sensitivity (%)	Specificity (%)
VFA/SFA	0.640	0.572–0.707	<0.001	0.73	73.1	48.7
MA/SBW (cm^2^/kg)	0.690	0.626–0.753	<0.001	1.55	88.0	46.0
Age (years)	0.666	0.600–0.732	<0.001	56.50	88.9	38.0
Albumin (g/L)	0.600	0.530–0.670	0.006	29.75	64.8	55.3
APACHE Ⅱ score	0.700	0.637–0.763	<0.001	14.50	65.7	64.7
Lactate (mmol/L)	0.693	0.629–0.757	<0.001	2.05	70.4	64.0

APACHE: Acute Physiology and Chronic Health Evaluation; ARDS: Acute respiratory distress syndrome; AUC: Area under the curve; CI: Confidence interval; MA: Muscle area; SBW: Standard body weight; SFA: Subcutaneous fat area; VFA: Visceral fat area.

For the ratio of VFA/SFA, the optimal cut-off was identified as 0.73, yielding a sensitivity of 73.1% and a specificity of 48.7%. The associated area under the curve (AUC) was 0.640 (95% CI 0.572–0.707, *P* <0.001). For the metric of MA/SBW, a threshold of 1.55 cm^2^/kg was optimal, demonstrating a sensitivity of 88.0% and a specificity of 46.0%. The AUC for this parameter was 0.690 (95% CI 0.626–0.753, *P* <0.001).The age threshold that presented the best performance was 56.50 years, with a sensitivity of 88.9% and specificity of 38.0%. Its AUC was 0.666 (95% CI 0.600–0.732, *P* <0.001). For albumin, a threshold of 29.75 g/L was determined optimal, reflecting a sensitivity of 64.8% and specificity of 55.3%. The associated AUC for albumin was 0.600 (95% CI 0.530–0.670, *P*=0.006). The APACHE II score presented an optimal threshold at 14.50, achieving a sensitivity of 65.7% and a specificity of 64.7%. Its AUC was calculated as 0.700 (95% CI 0.637–0.763, *P* <0.001). For lactate, the most suitable threshold was determined at 2.05 mmol/L, with a sensitivity of 70.4% and specificity of 64.0%. The AUC for this predictor was 0.693 (95% CI 0.629–0.757, *P* <0.001).

#### Comparison of clinical characteristics of patients with ARDS grouped by VFA/SFA levels

Patients with ARDS were categorized based on the pivotal threshold for the VFA to SFA ratio, as determined from the ROC analysis: patients with a ratio below 0.73 and those with a ratio of 0.73 or above. Within the original cohort, 100 patients registered a ratio of less than 0.73, while 158 patients presented a ratio of 0.73 or higher (Supplementary Table 1). To address potential confounding factors, a 1:1 PSM was performed, accounting for variables such as age, sex, BMI, underlying diseases, and laboratory markers immediately upon ICU admission. After the matching procedure, each group consisted of 68 patients. Following this adjustment, the two groups displayed no notable differences in demographic details, underlying health conditions, or laboratory results. This indicates a successful alignment of baseline characteristics, thus making the outcomes between the groups more comparable. Importantly, patients with a VFA to SFA ratio of ≥0.73 exhibited a less favorable prognosis than those with a ratio of <0.73, as evidenced by a significantly elevated in-hospital mortality rate (54.4% *vs*. 25.0%, *P*=0.001) (Table 4).

**Table 4:** Comparison of clinical characteristics of patients with ARDS grouped by VFA/SFA levels.

Variables	VFA/SFA <0.73 (*n*=68)	VFA/SFA ≥0.73 (*n*=68)	Statistics	*P* values
Age (years)	64.38±20.10	64.43±17.34	*t* = 0.020	0.984
Male	44 (64.7)	44 (64.7)	χ² = 0	1.000
BMI (kg/m^2^)	23.48±4.69	23.26±4.00	*t* = 0.595	0.553
Smoking history	22 (32.4)	16 (23.5)	χ² = 0.963	0.327
Underlying diseases				
Hypertension	40 (58.8)	34 (50.0)	χ² = 0.735	0.391
Diabetes	17 (25.0)	12 (17.6)	χ² = 0.944	0.332
Coronary heart disease	6 (8.8)	4 (5.9)	-	0.744
Hyperlipidemia	3 (4.4)	4 (5.9)	-	1.000
COPD	6 (8.8)	7 (10.3)	χ² = 0.094	1.000
ARDS classification upon ICU admission			χ² = 1.890	0.389
Mild	18 (26.5)	20 (29.4)		
Moderate	31 (45.6)	36 (52.9)		
Severe	19 (27.9)	12 (17.6)		
APACHE Ⅱ score	14.09±5.36	14.60±5.82	*t* = 0.531	0.597
SOFA score	7.59±3.08	7.15±3.47	*t* = 0.839	0.402
Laboratory indicators upon ICU admission				
Neutrophil count (× 10^9^/L)	10.54 (5.90, 14.85)	10.10 (6.49, 13.69)	*U* = 2150.5	0.586
Platelet count (× 10^9^/L)	168.42 (110.24, 227.80)	188.26 (112.23, 256.99)	*U* = 2018.0	0.225
Hemoglobin (g/L)	113.32±29.33	108.78±25.75	*t* = 1.110	0.268
Albumin (g/L)	30.28±5.87	29.89±6.14	*t* = 0.403	0.687
AST (U/L)	38.70 (23.75, 87.95)	36.50 (25.75, 84.25)	*U* = 2218.0	0.673
ALT (U/L)	27.00 (17.53, 50.00)	22.00 (15.00, 49.35)	*U* = 2060.5	0.249
Arterial blood gas upon ICU admission				
PaO_2_/FiO_2_ (mmHg)	165.83 (116.53, 214.48)	182.13 (116.25, 253.07)	*U* = 1953.0	0.065
Lactate (mmol/L)	1.50 (1.10, 3.15)	2.00 (1.23, 2.88)	*U* = 2097.5	0.255
Mechanical ventilation duration (days)	8.00 (4.00, 16.00)	8.00 (5.00, 14.75)	*U* = 2180.0	0.605
ICU length of stay (days)	18.36 (8.98, 27.64)	15.68 (8.38, 23.03)	*U* = 2048.5	0.241
Outcome				
In-hospital mortality rate	17 (25.0)	37 (54.4)	χ² = 11.310	0.001

Data are presented as mean±standard deviation, median (Q_1_, Q_3_) or *n* (%). ALT: Alanine aminotransferase; APACHE: Acute Physiology and Chronic Health Evaluation; ARDS: Acute respiratory distress syndrome; AST: Aspartate aminotransferase; BMI: Body mass index; COPD: Chronic obstructive pulmonary disease; FiO_2_: Fraction of inspired oxygen; ICU: Intensive care unit; MA: Muscle area; PaO_2_: Partial pressure of oxygen in arterial blood; SBW: Standard body weight; SFA: Subcutaneous fat area; SOFA: Sequential Organ Failure Assessment; VFA: Visceral fat area; -: Not available.

#### Comparison of clinical characteristics of patients with ARDS grouped by MA/SBW levels

Based on the MA/SBW critical value determined through ROC curve analysis, patients with ARDS were categorized into two patient groups: those with MA/SBW <1.55 cm²/kg (*n*=174) and those with MA/SBW ≥1.55 cm²/kg (*n*=84). However, an imbalance was noted in the baseline characteristics of these two groups (Supplementary Table 2). To rectify this potential skew, a 1:1 PSM was undertaken, accounting for variables including age, sex, BMI, underlying health conditions, and laboratory measures. Both groups, distinguished by MA/SBW ratios of either <1.55 cm^2^/kg or ≥1.55 cm^2^/kg, comprised 63 patients (Table 5). Remarkably, post-matching comparisons revealed no significant deviations between the two groups in demographics, underlying diseases, APACHE II and SOFA scores, or laboratory evaluations, underscoring a harmonized baseline and ensuring outcome comparability. After this adjustment, patients with a MA/SBW ratio of ≥1.55 cm^2^/kg exhibited a significantly lower in-hospital mortality rate compared with those in the <1.55 cm^2^/kg group (19.0% *vs*. 52.4%, *P* <0.001).

**Table 5:** Comparison of clinical characteristics of patients with ARDS grouped by MA/SBW levels.

Variables	MA/SBW <1.55 cm^2^/kg (*n*=63)	MA/SBW ≥1.55 cm^2^/kg (*n*=63)	Statistics	*P* values
Age (years)	64.60±18.29	63.78±18.10	*t* = 0.264	0.792
Male	48 (76.2)	44 (69.8)	χ² = 0.407	0.523
BMI (kg/m^2^)	24.41±3.29	24.37±3.37	*t* = 0.310	0.758
Smoking history	21 (33.3)	21 (33.3)	χ² = 0	1.000
Underlying diseases				
Hypertension	37 (58.7)	37 (58.7)	χ² = 0	1.000
Diabetes	15 (23.8)	16 (25.4)	χ² = 0.042	0.838
Coronary heart disease	6 (9.5)	3 (4.8)	-	0.491
Hyperlipidemia	2 (3.2)	2 (3.2)	-	1.000
COPD	5 (7.9)	5 (7.9)	χ² = 0	1.000
ARDS classification upon ICU admission			χ² = 1.202	0.547
Mild	16 (25.4)	20 (31.7)		
Moderate	32 (50.8)	33 (52.4)		
Severe	17 (27.0)	12 (19.0)		
APACHE Ⅱ score	14.57±5.48	14.17±5.31	*t* = 0.407	0.686
SOFA score	7.43±3.27	8.67±3.62	*t*=-1.846	0.066
Laboratory indicators upon ICU admission				
Neutrophil count (× 10^9^/L)	10.74±6.88	11.06±6.51	*t*=-0.288	0.774
Platelet count (× 10^9^/L)	152.00 (95.00, 226.00)	145.00 (66.00, 218.00)	*U* = 1880.5	0.318
Hemoglobin (g/L)	110.98±31.42	112.32±28.02	*t*=-0.229	0.819
Albumin (g/L)	30.26±5.98	30.58±6.35	*t*=-0.284	0.777
AST (U/L)	34.00 (24.00, 63.00)	44.60 (24.00, 70.00)	*U* = 1920.0	0.649
ALT (U/L)	25.00 (14.90, 59.00)	32.30 (21.60, 63.00)	*U* = 1792.0	0.357
Arterial blood gas upon ICU admission				
PaO_2_/FiO_2_ (mmHg)	165.45 (118.78, 247.50)	168.57 (115.85, 219.05)	*U* = 1950.5	0.805
Lactate (mmol/L)	2.00 (1.40, 3.30)	1.50 (1.10, 3.00)	*U* = 1903.0	0.568
Mechanical ventilation duration (days)	8.00 (5.00, 18.00)	8.00 (4.00, 14.00)	*U* = 1981.0	0.816
ICU length of stay (days)	14.00 (7.00, 23.00)	14.00 (8.00, 25.00)	*U* = 1958.0	0.499
Outcome				
In-hospital mortality	33 (52.4)	12 (19.0)	χ² = 13.410	<0.001

Data are presented as mean±standard deviation, median (Q_1_, Q_3_) or *n* (%). ALT: Alanine aminotransferase; APACHE: Acute Physiology and Chronic Health Evaluation; ARDS: Acute respiratory distress syndrome; AST: Aspartate aminotransferase; BMI: Body mass index; COPD: Chronic obstructive pulmonary disease; FiO_2_: Fraction of inspired oxygen; ICU: Intensive care unit; MA: Muscle area; PaO_2_: Partial pressure of oxygen in arterial blood; SBW: Standard body weight; SFA: Subcutaneous fat area; SOFA: Sequential Organ Failure Assessment; VFA: Visceral fat area; -: Not available.

#### Survival analysis with Kaplan–Meier curves for patients with ARDS

**Fig. 3:**
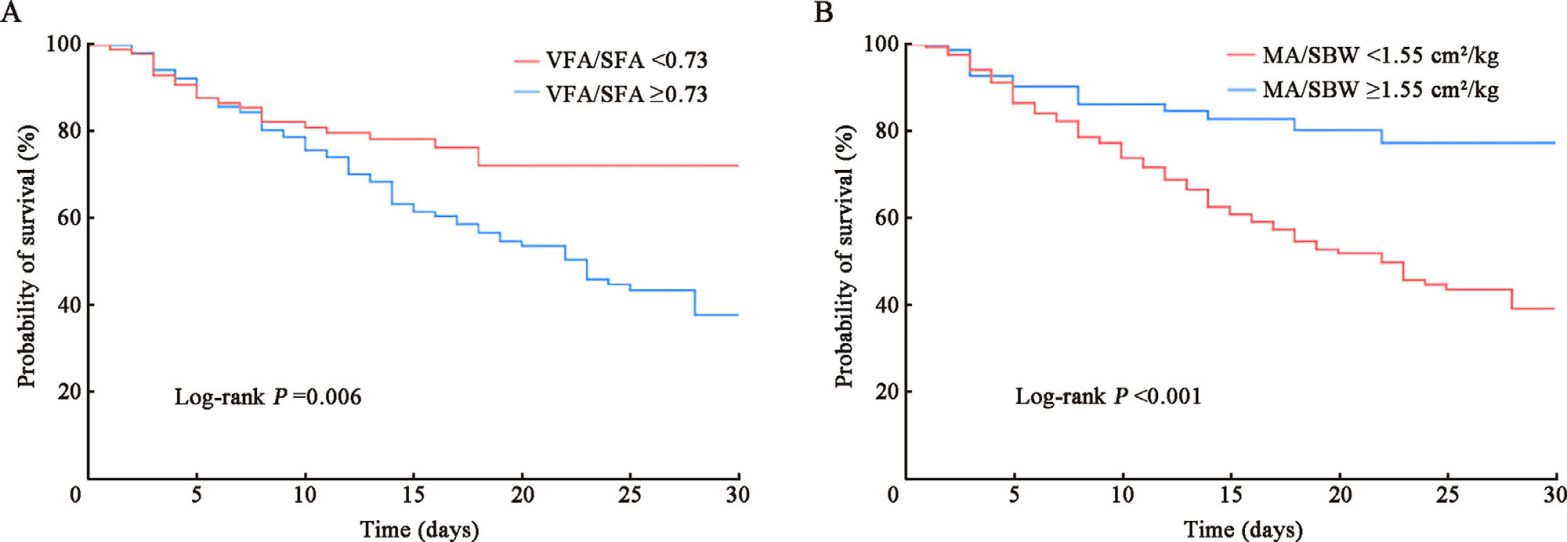
Kaplan–Meier survival curves for patients with ARDS. (A) Kaplan-Meier survival curve for the VFA/SFA ratio; (B) Kaplan-Meier survival curve for the MA/SBW ratio. ARDS: Acute respiratory distress syndrome; MA: Muscle area; SBW: Standard body weight; SFA: Subcutaneous fat area; VFA: Visceral fat area.

As illustrated in Fig. 3, the Kaplan–Meier survival curves for patients with ARDS indicate that individuals with a VFA/SFA ratio <0.73 exhibited a superior survival rate during their ICU stay in comparison with those with a ratio ≥0.73 (log-rank *P*=0.006). Similarly, patients with a MA/SBW ratio ≥1.55 cm^2^/kg demonstrated a notably higher survival rate throughout their ICU stay compared with those having a ratio <1.55 cm^2^/kg (log-rank *P* <0.001).

### Relationship between T12 CT level fat areas and MA and the prognosis of pulmonary-origin ARDS

#### Comparison of clinical characteristics in patients with pulmonary-origin ARDS stratified by different outcomes

From a cohort of 223 patients satisfying the inclusion and exclusion criteria, 170 were diagnosed with pulmonary-origin ARDS, as outlined in Table 6. These patients were subsequently divided into two groups based on survival outcomes: the survival group, with 87 individuals, and the death group, comprising 83 individuals. Upon a thorough comparison of clinical features and prognostic markers between these two categories, several significant differences surfaced. Notably, the death group had a significantly higher median age relative to the survival group (*P*=0.011). With regard to laboratory findings upon ICU admission, the death group manifested notably lower albumin levels (*P*=0.005) and elevated APACHE II scores and lactate levels compared to the survival group (*P* <0.001). In terms of chest fat and muscle metrics, the death group exhibited decreased values for both SFA and the SFA/SBW ratio (*P*=0.004, *P*=0.003, respectively). Similarly, they exhibited a decreased MA and a reduced MA/SBW ratio (*P*=0.001, *P* <0.001, respectively). Moreover, a significantly higher VFA/SFA ratio was observed in the death group (*P* <0.001). Yet, no significant disparities were identified for VFA, VFA/SBW ratio, or the ratio of thoracic transverse diameter to anteroposterior diameter between the groups (*P*=0.474, *P*=0.450, *P*=0.258, respectively). Finally, sex, BMI, smoking status, pre-existing medical conditions, SOFA scores, other laboratory metrics, and oxygenation indexes did not demonstrate any statistically significant differences between the survival and death groups.

**Table 6:** Comparison of clinical characteristics of patients with pulmonary-origin ARDS stratified by different outcomes.

Variables	Survival group (*n*=87)	Death group (*n*=83)	Statistics	*P* values
Age (years)	71.00 (59.00, 79.00)	76.00 (67.00, 84.00)	*U* = 2814.0	0.011
Male	59 (67.8)	59 (71.1)	χ² = 0.213	0.644
BMI (kg/m^2^)	22.86 (20.76, 25.25)	22.20 (19.98, 25.46)	*U* = 3240.0	0.288
Smoking history	26 (29.9)	22 (26.5)	χ² = 0.238	0.625
Underlying diseases				
Hypertension	50 (57.5)	54 (65.1)	χ² = 1.028	0.310
Diabetes	22 (25.3)	22 (26.5)	χ² = 0.033	0.856
Coronary heart disease	6 (6.9)	7 (8.4)	χ² = 0.144	0.706
Hyperlipidemia	1 (1.1)	2 (2.4)	-	0.614
COPD	10 (11.5)	8 (9.6)	χ² = 0.168	0.694
Etiology of pulmonary-origin ARDS				
Pulmonary infection	80 (92.0)	82 (98.8)	χ² = 3.855	0.050
Aspiration of gastric contents	4 (4.6)	0 (0)	-	1.000
Inhalation of irritant gases	1 (1.1)	0 (0)	-	1.000
Drowning	2 (2.3)	1 (1.2)	-	1.000
ARDS classification upon ICU admission			χ² = 3.012	0.221
Mild	28 (32.2)	22 (26.5)		
Moderate	40 (46.0)	33 (39.8)		
Severe	19 (21.8)	28 (33.7)		
APACHE Ⅱ score	13.00 (9.00, 16.00)	15.00 (13.00, 19.00)	*U* = 2352.0	<0.001
SOFA score	6.00 (5.00, 9.00)	7.00 (5.00, 10.00)	*U* = 3394.0	0.435
Laboratory indicators upon ICU admission				
Neutrophil count (× 10^9^/L)	9.40 (5.66, 13.66)	9.53 (5.96, 14.30)	*U* = 3441.0	0.475
Platelet count (× 10^9^/L)	173.00 (122.00, 232.00)	156.00 (89.00, 241.00)	*U* = 3195.0	0.190
Hemoglobin (g/L)	115.82±27.88	114.20±24.29	*t* = 0.398	0.689
Albumin (g/L)	31.30 (28.30, 35.90)	28.60 (24.60, 32.60)	*U* = 2970.5	0.005
AST (U/L)	33.20 (21.00, 60.00)	33.00 (25.00, 81.00)	*U* = 3425.5	0.289
ALT (U/L)	23.00 (14.00, 53.00)	27.00 (17.00, 56.00)	*U* = 3243.5	0.180
Arterial blood gases upon ICU admission				
PaO_2_/FiO_2_ (mmHg)	163.03 (119.62, 228.10)	160.80 (90.48, 242.42)	*U* = 3348.5	0.317
Lactate (mmol/L)	1.60 (1.20, 2.80)	2.60 (1.50, 3.70)	*U* = 2600.5	<0.001
Chest fat and muscle area				
SFA (cm^2^)	68.53 (48.27, 95.79)	51.58 (27.13, 88.78)	*U* = 2918.0	0.004
VFA (cm^2^)	54.00 (29.66, 92.58)	63.13 (28.18, 105.81)	*U* = 3444.5	0.474
MA (cm^2^)	85.21 (64.21, 102.30)	77.49 (61.39, 84.37)	*U* = 2750.0	0.001
Chest fat and muscle area adjusted for standard weight				
SFA/SBW (cm^2^/kg)	1.09 (0.81, 1.54)	0.83 (0.46, 1.42)	*U* = 2874.0	0.003
VFA/SBW (cm^2^/kg)	0.97 (0.48, 1.50)	1.05 (0.48, 1.71)	*U* = 3451.5	0.450
MA/SBW (cm^2^/kg)	1.43 (1.16, 1.70)	1.26 (1.04, 1.40)	*U* = 2537.0	<0.001
VFA/SFA	0.72 (0.47, 1.11)	1.07 (0.65, 1.72)	*U* = 2723.0	<0.001
Thoracic transverse diameter/anteroposterior diameter	2.14 (1.93, 2.37)	2.23 (1.98, 2.38)	*U* = 3284.5	0.258

Data are presented as mean±standard deviation, median (Q_1_, Q_3_) or *n* (%). ALT: Alanine aminotransferase; APACHE: Acute Physiology and Chronic Health Evaluation; ARDS: Acute respiratory distress syndrome; AST: Aspartate aminotransferase; BMI: Body mass index; COPD: Chronic obstructive pulmonary disease; FiO_2_: Fraction of inspired oxygen; ICU: Intensive care unit; MA: Muscle area; PaO_2_: Partial pressure of oxygen in arterial blood; SBW: Standard body weight; SFA: Subcutaneous fat area; SOFA: Sequential Organ Failure Assessment; VFA: Visceral fat area; -: Not available.

#### Univariable and multivariable logistic regression analysis of mortality in patients with pulmonary-origin ARDS

As presented in Table 7, we utilized univariable logistic regression to pinpoint prognostically significant variables for pulmonary-origin ARDS. Those achieving a *P*-value of less than 0.1 in the univariable analysis were further assessed using multivariable logistic regression. The multivariable findings revealed that age (OR=1.030, 95% CI 1.001–1.060, *P*=0.042), APACHE II score (OR=1.139, 95% CI 1.050–1.235, *P*=0.002), lactate concentrations (OR=1.256, 95% CI 1.044–1.512, *P*=0.016), and the VFA/SFA ratio (OR=2.397, 95% CI 1.259–4.564, *P*=0.008) stood out as distinct predictors of increased mortality risk in patients with pulmonary-origin ARDS. In contrast, serum albumin concentrations (OR=0.937, 95% CI 0.880–0.999, *P*=0.046) and the MA/SBW ratio (OR=0.060, 95% CI 0.014–0.262, *P* <0.001) were identified as independent protective factors, correlating with a reduced likelihood of death in this cohort.

**Table 7:** Univariable and multivariable logistic regression analysis of mortality in patients with pulmonary-origin ARDS.

Variables	Univariable regression	*P* values	Multivariable regression	*P* values
	OR (95% CI)		OR (95% CI)	
Age (years)	1.032 (1.010–1.056)	0.005	1.030 (1.001–1.060)	0.042
APACHE II score	1.160 (1.080–1.246)	<0.001	1.139 (1.050–1.235)	0.002
Albumin (g/L)	0.942 (0.895–0.992)	0.023	0.937 (0.880–0.999)	0.046
Lactate (mmol/L)	1.249 (1.052–1.483)	0.011	1.256 (1.044–1.512)	0.016
SFA/SBW (cm^2^/kg)	0.548 (0.350–0.858)	0.009	0.570 (0.340–0.956)	0.032
VFA/SFA	2.066 (1.294–3.297)	0.002	2.397 (1.259–4.564)	0.008
MA/SBW (cm^2^/kg)	0.152 (0.056–0.417)	<0.001	0.060 (0.014–0.262)	<0.001

APACHE: Acute Physiology and Chronic Health Evaluation; ARDS: Acute respiratory distress syndrome; CI: Confidence interval; MA: Muscle area; OR: Odds ratio; SBW: Standard body weight; SFA: Subcutaneous fat area; VFA: Visceral fat area.

#### ROC curve analysis of prognosis in pulmonary-origin ARDS, and analysis of critical values, sensitivity, and specificity of ARDS prognostic factors

**Fig. 4:**
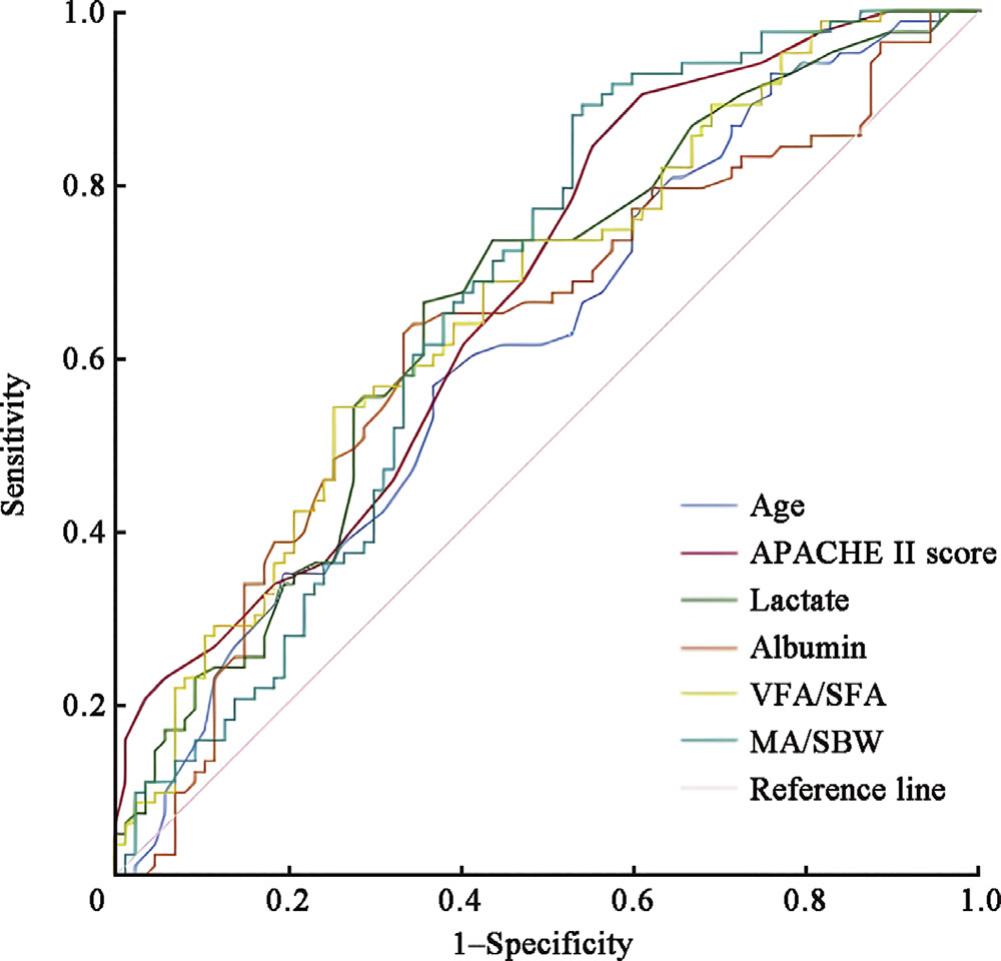
ROC curve for predicting mortality in pulmonary-origin ARDS. APACHE: Acute Physiology and Chronic Health Evaluation; ARDS: Acute respiratory distress syndrome; MA: Muscle area; ROC: Receiver operating characteristic; SBW: Standard body weight; SFA: Subcutaneous fat area; VFA: Visceral fat area.

As shown in Fig. 4 and detailed in Table 8, we utilized ROC curves to ascertain the optimal thresholds for predicting mortality in patients with pulmonary-origin ARDS.

**Table 8:** Analysis of critical values for mortality predictive indicators in patients with pulmonary-origin ARDS.

Variables	AUC	95% CI	*P* values	Optimal cut-off values	Sensitivity (%)	Specificity (%)
VFA/SFA	0.665	0.584–0.750	<0.001	1.01	74.7	54.2
MA/SBW (cm^2^/kg)	0.668	0.586–0.750	<0.001	1.48	89.2	46.0
Age (years)	0.613	0.529–0.697	0.011	74.50	56.6	63.2
Albumin (g/L)	0.625	0.540–0.710	0.005	29.75	63.9	65.5
APACHE II Score	0.673	0.593–0.753	<0.001	10.50	90.4	39.1
Lactate (mmol/L)	0.660	0.578–0.741	<0.001	2.05	66.3	64.4

APACHE: Acute Physiology and Chronic Health Evaluation; ARDS: Acute respiratory distress syndrome; AUC: Area under the curve; CI: Confidence interval; MA: Muscle area; SBW: Standard body weight; SFA: Subcutaneous fat area; VFA: Visceral fat area.

Pertinently, the most favorable threshold for the VFA/SFA ratio was at 1.01, exhibiting a sensitivity of 74.7% and a specificity of 54.2%. This threshold corresponded to an AUC value of 0.665 (95% CI 0.584–0.750, *P* <0.001). For the MA/SBW ratio, the threshold was pinpointed at 1.48 cm^2^/kg, showing a sensitivity of 89.2%, a specificity of 46.0%, and an AUC of 0.668 (95% CI 0.586–0.750, *P* <0.001). Furthermore, the age threshold was determined to be 74.50 years, demonstrating a sensitivity of 56.6% and a specificity of 63.2%, and was associated with an AUC of 0.613 (95% CI 0.529–0.697, *P*=0.011). Regarding albumin levels, a threshold of 29.75 g/L was deemed optimal, reflecting a sensitivity of 63.9% and a specificity of 65.5%, and yielding an AUC of 0.625 (95% CI 0.540–0.710, *P*=0.005). Additionally, the APACHE II score's pivotal threshold was identified as 10.50, with a sensitivity of 90.4% and a specificity of 39.1%, and an AUC of 0.673 (95% CI 0.593–0.753, *P* <0.001). For lactate levels, a threshold of 2.05 mmol/L was found most fitting, offering a sensitivity of 66.3% and a specificity of 64.4%, with an AUC of 0.660 (95% CI 0.578–0.741, *P* <0.001).

#### Comparison of clinical characteristics of patients with pulmonary-origin ARDS grouped by VFA/SFA levels

Based on the pivotal threshold identified from the ROC curve for the VFA/SFA ratio, patients with pulmonary-origin ARDS were stratified into two cohorts: those with a ratio of less than 1.01 (*n*=102) and those at or exceeding 1.01 (*n*=68). Initially, there was a noticeable imbalance in the baseline characteristics between these two cohorts (Supplementary Table 3). To counteract potential biases, we instituted a 1:1 PSM, adjusting for variables such as age, sex, BMI, existing comorbidities, and laboratory metrics upon ICU admission. This process, outlined in Table 9, resulted in a pair-matched set of 46 patients for each group. Post-PSM, the cohorts demonstrated no discernible statistical disparities in demographics, prevalent comorbidities, or laboratory evaluations. This alignment underscores a harmonized baseline, reinforcing the comparability of outcomes. Strikingly, our data indicated that patients characterized by a VFA/SFA ratio of ≥1.01 experienced a more adverse prognosis, exemplified by a notably elevated in-hospital mortality rate in contrast to those within the <1.01 bracket (69.6% *vs*. 39.1%, *P*=0.004).

**Table 9:** Comparison of clinical characteristics of patients with pulmonary-origin ARDS grouped by VFA/SFA levels.

Variables	VFA/SFA<	VFA/SFA≥	Statistics	*P* values
	1.01 (*n*=46)	1.01 (*n*=46)		
Age (years)	76.22±9.78	76.11±9.40	*t* = 0.261	0.794
Male	40 (87.0)	40 (87.0)	χ² = 0	1.000
BMI (kg/m^2^)	22.57±3.71	22.35±3.52	*t* = 0.274	0.785
Smoking history	19 (41.3)	12 (26.1)	χ² = 1.915	0.167
Underlying diseases				
Hypertension	33 (71.7)	34 (73.9)	χ² = 0.046	0.831
Diabetes	14 (30.4)	12 (26.1)	χ² = 0.238	0.626
Coronary heart disease	6 (13.0)	3 (6.5)	-	0.485
Hyperlipidemia	1 (2.2)	0 (0)	-	1.000
COPD	6 (13.0)	5 (10.9)	χ² = 0.102	0.749
ARDS classification upon ICU admission			χ² = 0.180	0.914
Mild	15 (32.6)	16 (34.8)		
Moderate	21 (45.7)	19 (41.3)		
Severe	10 (21.7)	11 (23.9)		
APACHE II score	14.35±4.15	16.30±6.12	*t*=-1.798	0.076
SOFA score	7.22±3.25	7.54±3.45	*t*=-0.458	0.649
Laboratory indicators upon ICU admission				
Neutrophil count (× 10^9^/L)	11.81 (6.53, 16.69)	10.82 (6.92, 14.69)	*U*=1288.0	0.068
Platelet count (× 10^9^/L)	196.80 (129.03, 265.98)	186.61 (105.29, 260.12)	*U* = 986.0	0.675
Hemoglobin (g/L)	120.96±28.54	114.15±27.00	*t* = 1.316	0.194
Albumin (g/L)	31.05 (28.60, 35.20)	28.85 (24.43, 36.00)	*U* =1324.0	0.035
AST (U/L)	33.00 (22.00, 58.75)	29.00 (19.40, 82.95)	*U* = 986.5	0.462
ALT (U/L)	24.50 (15.60, 41.50)	21.90 (12.65, 60.35)	*U* = 988.0	0.750
Arterial blood gas upon ICU admission				
PaO_2_/FiO_2_ (mmHg)	161.96 (97.10, 219.48)	171.66 (83.91, 250.95)	*U* = 989.0	0.996
Lactate (mmol/L)	2.20 (1.40, 3.35)	2.45 (1.50, 3.63)	*U* = 987.0	0.631
Mechanical ventilation duration (days)	6.00 (4.00, 14.25)	9.50 (6.00, 17.00)	*U* = 973.5	0.115
ICU length of stay (days)	12.50 (5.75, 25.00)	12.00 (7.75, 22.25)	*U* = 998.5	0.983
Outcome				
In-hospital mortality rate	18 (39.1)	32 (69.6)	χ² = 8.341	0.004

Data are presented as mean±standard deviation, median (Q_1_, Q_3_) or *n* (%). ALT: Alanine aminotransferase; APACHE: Acute Physiology and Chronic Health Evaluation; ARDS: Acute respiratory distress syndrome; AST: Aspartate aminotransferase; BMI: Body mass index; COPD: Chronic obstructive pulmonary disease; FiO_2_: Fraction of inspired oxygen; ICU: Intensive care unit; MA: Muscle area; PaO_2_: Partial pressure of oxygen in arterial blood; SBW: Standard body weight; SFA: Subcutaneous fat area; SOFA: Sequential Organ Failure Assessment; VFA: Visceral fat area; -: Not available.

#### Comparison of clinical characteristics of patients with pulmonary-origin ARDS grouped according to MA/SBW levels

Patients with pulmonary-origin ARDS were stratified into two groups using the cut-off value derived from the ROC curve for the MA/SBW ratio. These groups were delineated as patients with a MA/SBW ratio below 1.48 cm²/kg (*n*=121) and those with a ratio of 1.48 cm²/kg or above (*n*=49). To address the imbalance in baseline characteristics between the two groups of patients, we conducted a 1:1 PSM (Supplementary Table 4). This process culminated in a harmonized cohort delineated in Table 10, with each subgroup containing 44 patients. Following this rigorous matching process, a comparative analysis of the baseline demographics, pre-existing conditions, and laboratory measures revealed an absence of statistically significant differences between the groups. This affirms a balanced baseline and consistent potential outcomes across both groups. Crucially, post-PSM analysis indicated that patients with an MA/SBW ratio of ≥1.48 cm²/kg manifested a considerably reduced in-hospital mortality rate when juxtaposed against their peers with a ratio <1.48 cm²/kg (18.2% *vs*. 54.5%, *P* <0.001), hinting at a superior prognosis for the former subgroup.

**Table 10:** Comparison of clinical characteristics of patients with pulmonary-origin ARDS grouped according to MA/SBW levels.

Variables	MA/SBW < 1.48 cm^2^/kg (*n*=44)	MA/SBW ≥1.48 cm^2^/kg (*n*=44)	Statistics	*P* values
Age (years)	72.86±12.57	72.20±14.65	*t* = 0.232	0.816
Male	30 (68.2)	30 (68.2)	χ² = 0	1.000
BMI (kg/m^2^)	24.22 (22.03, 26.41)	24.13 (21.80, 26.32)	*t* = 0.256	0.800
Smoking history	14 (31.8)	16 (36.4)	χ² = 0.041	0.839
Underlying diseases				
Hypertension	26 (59.1)	32 (72.7)	χ² = 1.139	0.286
Diabetes	15 (34.1)	13 (29.5)	χ² = 0.062	0.804
Coronary heart disease	1 (2.3)	6 (13.6)	-	0.110
Hyperlipidemia	1 (2.3)	2 (4.5)	-	1.000
COPD	2 (4.5)	5 (11.4)	-	0.430
ARDS classification upon ICU admission			χ² = 1.763	0.414
Mild	8 (18.2)	12 (27.3)		
Moderate	26 (59.1)	20 (45.5)		
Severe	10 (22.7)	12 (27.3)		
APACHE II score	14.89±4.77	13.66±4.12	*t* = 1.186	0.237
SOFA score	7.55±3.14	7.50±3.05	*t* = 0.064	0.949
Laboratory indicators upon ICU admission				
Neutrophil count (× 10^9^/L)	9.87 (5.49, 13.93)	10.44 (6.93, 13.93)	*U* = 900.0	0.764
Platelet count (× 10^9^/L)	153.00 (104.75, 223.00)	158.50 (93.00, 216.00)	*U* = 950.0	0.636
Hemoglobin (g/L)	112.00±27.11	115.68±28.35	*t* = 0.624	0.535
Albumin (g/L)	30.35±4.97	31.31±6.20	*t* = 0.787	0.435
AST (U/L)	33.00 (24.40, 65.50)	31.50 (20.25, 54.00)	*U* = 850.0	0.111
ALT (U/L)	24.00 (14.25, 60.28)	27.95 (15.00, 47.00)	*U* = 980.0	0.657
Arterial blood gas upon ICU admission				
PaO_2_/FiO_2_ (mmHg)	181.90±85.82	155.16±68.35	*t* = 1.510	0.136
Lactate (mmol/L)	1.90 (1.33, 2.70)	1.50 (1.23, 2.70)	*U* = 990.0	0.945
Mechanical ventilation duration (days)	6.50 (4.00, 13.50)	8.50 (5.00, 20.00)	*U* = 940.0	0.135
ICU length of stay (days)	10.00 (5.00, 24.00)	14.00 (7.25, 25.50)	*U* = 950.0	0.246
Outcome				
In-hospital mortality rate	24 (54.5)	8 (18.2)	χ² = 10.911	<0.001

Data are presented as mean±standard deviation, median (Q_1_, Q_3_) or *n* (%). ALT: Alanine aminotransferase; APACHE: Acute Physiology and Chronic Health Evaluation; ARDS: Acute respiratory distress syndrome; AST: Aspartate aminotransferase; BMI: Body mass index; COPD: Chronic obstructive pulmonary disease; FiO_2_: Fraction of inspired oxygen; ICU: Intensive care unit; MA: Muscle area; PaO_2_: Partial pressure of oxygen in arterial blood; SBW: Standard body weight; SOFA: Sequential Organ Failure Assessment; -: Not available.

#### Kaplan–Meier survival curve analysis of patients with pulmonary-origin ARDS

**Fig. 5:**
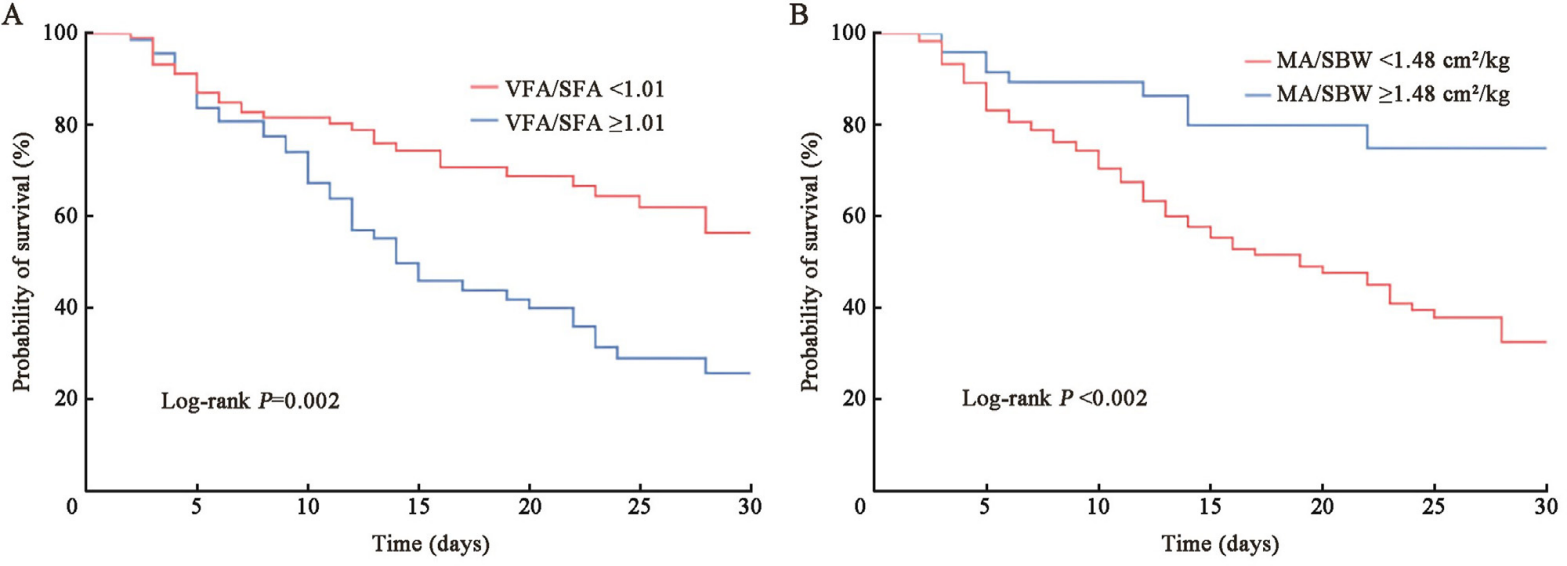
Kaplan–Meier survival curves of patients with pulmonary-origin ARDS. (A) Kaplan-Meier survival curve for the VFA/SFA ratio; (B) Kaplan-Meier survival curve for the MA/SBW ratio. ARDS: Acute respiratory distress syndrome; MA: Muscle area; SBW: Standard body weight; SFA: Subcutaneous fat area; VFA: Visceral fat area.

Fig. 5 depicts the results of Kaplan–Meier survival curve analysis among patients with pulmonary-origin ARDS. The analysis reveals that patients with a VFA/SFA ratio <1.01 exhibited a superior survival rate during their ICU hospitalization compared with those with a ratio ≥1.01 (log-rank *P*=0.002). Furthermore, patients with a MA/SBW ratio ≥1.48 cm²/kg demonstrate a notably higher survival rate during ICU hospitalization than their counterparts with a ratio <1.48 cm²/kg (log-rank *P*<0.001).

## Discussion

This study investigated the association between ARDS prognosis and various ratios at the T12 CT level: VFA/SFA, SFA/SBW, VFA/SBW, and MA/SBW. Furthermore, we delved deeply into other potential risk factors that might influence the prognosis of ARDS, validating their correlation with the prognosis of pulmonary-origin ARDS. Our findings suggest that the ratio of VFA/SFA at the T12 level, age, APACHE II score, and lactate are independent risk factors for ARDS mortality, while a higher MA/SBW ratio and albumin exert protective effects.

Through multivariable logistic regression analysis, our study identified an elevated ratio of VFA/SFA at the T12 level as an independent risk factor for mortality in patients with ARDS. Previous in-depth research on the relationship between abdominal fat and ARDS prognosis was conducted by the West China Medical School of Sichuan University. Based on abdominal CT scans at the L4–L5 level, there was a positive correlation between a higher ratio of VFA to SFA and increased mortality rates in patients with ARDS.^10^ Similarly, in our thoracic CT scans at the T12 level, we noticed a significant positive association between a higher VFA/SFA ratio and the risk of mortality in patients with ARDS. These findings collectively suggest a certain link between fat and ARDS prognosis, with the impact of fat on the prognosis potentially varying across different anatomical regions. This consistency across different studies reinforces the hypothesis that abdominal and perhaps thoracic adiposity plays a critical role in the prognosis of ARDS, suggesting a systemic influence of adipose tissue distribution on inflammatory and metabolic pathways involved in the disease's progression and outcome.

Adipose tissue, traditionally recognized as an energy storage organ, also functions as a dynamic endocrine entity. It actively secretes a multitude of bioactive substances, collectively termed adipokines, underscoring its multifaceted role in the body's physiological processes.^24^, ^25^, ^26^ Adipokines encompass pro-inflammatory cytokines such as tumor necrosis factor α (TNF-α), monocyte chemoattractant protein-1 (MCP-1), and interleukin-6 (IL-6), as well as anti-inflammatory factors like adiponectin and secreted frizzled-related protein 5 (SFRP5).^24^^,^^26^^,^^27^ The adipokines produced differ depending on the fat tissue location.^24^ Visceral and subcutaneous fats secrete unique adipokines, crucial for immune functions.^28^^,^^29^ Visceral fat predominantly releases pro-inflammatory cytokines like TNF-α and interleukins.^28^ Given that the primary pathophysiology of ARDS involves excessive inflammation and increased permeability of the alveolar–capillary barrier,^30^ the surge of inflammatory cytokines like TNF-α, IL-1, and IL-6 from visceral fat^28^ can further damage lung structures, leading to hypoxemia and respiratory failure, subsequently affecting prognosis.^4^^,^^31^ Subcutaneous fat primarily secretes the anti-inflammatory adipokine adipocyte complement-related 30 kDa protein,^28^ also known as ACRP30, a plasma adipocyte-derived factor.^32^^,^^33^ Adiponectin is pivotal in inhibiting pulmonary inflammation.^24^ In plasma, adiponectin levels inversely correlate with C-reactive protein (CRP) and IL-6 levels.^34^^,^^35^ Its anti-inflammatory properties may be associated with regulating macrophage functionality and phenotype, suppressing the nuclear factor kappa-B (NF-κB) signaling cascade, and diminishing TNF-α expression in macrophages triggered by lipopolysaccharide.^36^^,^^37^ Additionally, adiponectin prompts macrophages to release IL-10,^38^ an anti-inflammatory cytokine, which safeguards against systemic inflammation and enhances a positive prognosis. From a respiratory mechanics standpoint, increased thoracic subcutaneous fat might raise chest wall weight, leading to reduced respiratory system compliance. This implies that during mechanical ventilation, greater chest wall pressure must be overcome, resulting in reduced transpulmonary pressure, thereby mitigating the risk of ventilator-associated lung injury (VALI), further alleviating ARDS severity and decreasing mortality risk.^1^ Clinically, physicians may perceive obesity as indicating a worse prognosis, prompting early intervention like mobilization, pressure ulcer prevention, strict glycemic control, and meticulous mechanical ventilation management.^7^ In obese patients, clinicians may prefer “ultra-protective mechanical ventilation”, using tidal volumes below those of standard protective methods,^39^ a strategy shown to lessen lung injury and improve patient outcomes.^40^ The differences in pro-and anti-inflammatory factors secreted by visceral and subcutaneous fats, along with their impacts on respiratory mechanics, suggest that while visceral fat might exacerbate ARDS progression, subcutaneous fat might ameliorate ARDS inflammation, potentially improving its prognosis. This provides an explanation for the ratio of VFA/SFA serving as an independent risk factor for ARDS mortality. It also highlights the opportunity for targeted therapies and personalized ARDS management strategies, such as those modifying fat distribution or reducing visceral fat's inflammatory effects, offering new directions for treatment.

This study reveals that the ratio of MA/SBW at the T12 level serves as a protective indicator for the prognosis of ARDS. With an increase in this ratio, there is a reduction in the mortality risk of patients with ARDS. Thoracic muscles are closely associated with respiratory function. Animal studies indicate that autonomous respiratory muscle activity may improve gas exchange and lung ventilation.^41^ Larger thoracic muscle area indicates better lung ventilation, a decreased need for mechanical ventilation, reduced VALI, and a lower risk of mortality.^1^ In ARDS triggered by COVID-19, thoracic muscle metrics correlate with survival.^42^ Muscle depletion, linked to inflammation and lung injury, independently lowers survival rates.^42^^,^^43^ In patients with mild to moderate ARDS, muscle-aided spontaneous breathing improves oxygenation and reduces inflammation, fibrosis, and lung injury.^41^^,^^44^, ^45^, ^46^ While early literature regarded the diaphragm and intercostal muscles as the primary drivers of respiratory motion, with the thoracic spine playing only an auxiliary role,^47^^,^^48^ respiratory movement is highly reliant on the extension of the thoracic vertebrae, necessitating coordination with the erector spinae and other muscles. Their contraction results in the anterior convexity of the thoracic spine, enhancing the respiratory exchange surface area.^49^ Notably, the mobility linked with floating ribs at T12 is particularly significant. Research indicates that during deep breathing, there is a noticeable change in the distance from the T12 vertebra to the manubrium of the sternum,^49^ suggesting significant thoracic activity at the T12 level during respiratory motion. Thus, enlarged T12-level muscle area could enhance respiratory surface area, optimize lung ventilation, and improve ARDS prognosis.

This study confirms age as an independent risk factor for ARDS prognosis, consistent with prior research, underscoring that older age may worsen ARDS outcomes. A retrospective study^50^ explicitly stated that the prognosis for older patients was less favorable (*P*=0.04), verifying age as a critical determinant affecting the mortality rate of ARDS (OR=1.17; 95% CI 1.00–1.36; *P*=0.04). Additionally, a prospective multicenter study from the Washington state in the USA^51^ corroborated the detrimental effects of increasing age on prognosis (OR=1.25; 95% CI 1.14–1.37; *P*<0.05). Leyden and his team observed in a mouse model study of lung injury that mice aged 52–54 weeks had a significantly worse prognosis compared with those aged 8–12 weeks.^52^ The discrepancy may stem from age-related heightened inflammatory responses and pulmonary permeability, leading to severe edema, tissue damage, and elevated mortality risk.^52^, ^53^, ^54^, ^55^ Hence, this finding provides a reference for implementing stratified management in clinical practice based on the age of patients with ARDS.

The APACHE II system, integrating vital signs and multiple factors, is essential for assessing the severity and prognosis of critical illnesses. Studies^56^, ^57^, ^58^, ^59^ indicate that APACHE II scores can independently predict the mortality risk in patients with ARDS, with higher scores indicating worsened conditions and prognosis. Regarding the SOFA score, although some studies^60^^,^^61^ suggest its effectiveness in predicting the prognosis of ARDS caused by non-pulmonary sepsis and trauma, its correlation with prognosis in ARDS induced by pulmonary infection is not significant.^60^ This study found no conclusive predictive link between SOFA scores and ARDS mortality, likely because of the dominance of pulmonary infections over non-pulmonary causes in these ARDS cases.

Plasma lactate is a key metabolic indicator of tissue hypoxia, crucial for diagnosing and assessing the prognosis of diverse diseases. During intense exercise or certain pathologies causing insufficient oxygen or blood flow, tissue cells raise lactate levels in response to hypoxia.^62^ In ARDS, lung dysfunction causes hypoxemia, leading to increased lactate production and potential metabolic acidosis, a key predictor of severe post-traumatic lung injury.^63^ Furthermore, metabolic acidosis is closely associated with a high mortality rate in critically ill patients.^64^^,^^65^ Multiple studies indicate that higher lactate levels are an independent risk factor for ARDS mortality,^56^^,^^66^ consistent with our research findings.

The present study confirms the protective role of albumin in the prognosis of ARDS. Albumin performs multiple roles such as nutrient transport, osmotic pressure maintenance, acid–base balance, and inflammation regulation.^67^ In critically ill patients, increased capillary permeability because of inflammation and reduced synthesis of albumin in the acute phase may accelerate its consumption and loss.^68^ Low albumin levels can reflect both the nutritional status of patients and the activity of inflammation. Numerous studies have demonstrated a strong association between low albumin levels and poor prognosis in critically ill patients.^69^, ^70^, ^71^ Albumin plays a crucial role in maintaining the integrity of vascular endothelium and exerting anti-inflammatory effects.^72^^,^^73^ This may be a key factor in its protective role in ARDS prognosis. A systematic review has revealed that albumin supplementation may improve oxygenation in patients with ARDS.^74^ This improvement may stem from albumin's role in elevating colloid osmotic pressure, thereby diminishing alveolar and capillary permeability and lessening pulmonary edema. This finding offers crucial guidance for clinical practice, suggesting that albumin supplementation may effectively improve the prognosis of ARDS.

This study has its limitations. Primarily, by being retrospective, it may be subject to inherent biases typical of such a design. Nevertheless, we addressed some biases through 1:1 PSM, achieving basic consistency in baseline characteristics between groups and ensuring comparability of outcomes. Secondarily, the study had a limited sample size and focused subgroup analysis exclusively on pulmonary-origin ARDS, omitting subgroups for ARDS from other causes. Because of the limitations of the current research conditions, obtaining abdominal CT imaging data for patients with ARDS is more challenging than for chest CT, making it currently impossible for us to conduct a cross-sectional analysis of fat distribution in the chest and abdomen of patients to delineate differences in predictive superiority. Therefore, future studies will require larger participant groups and the incorporation of essential parameters, such as abdominal CT data, to fully understand the complex role of fat distribution in influencing ARDS outcomes. Additionally, it will be crucial to comprehensively evaluate the prognostic significance of fat distribution at the T12 level and muscle area in relation to ARDS outcomes.

In conclusion, VFA/SFA at the T12 level, age, APACHE II score, and lactate levels independently predict mortality in all patients with ARDS, including those with ARDS of pulmonary origin.

## Funding

This work was supported by a grant from Suzhou Science and Technology Project Plan (No. SZM2021006).

## Declaration of competing interest

The authors declared that they had no competing interests.

## Appendix Supplementary materials

Image, application 1
