# Supplementary material for: Association of ratios of visceral fat area/subcutaneous fat area and muscle area/standard body weight at T12 CT level with the prognosis of acute respiratory distress syndrome
Source: Chin Med J Pulm Crit Care Med. 2024 Jun 20;2(2):106–18. doi: 10.1016/j.pccm.2024.05.004 (PMC11332858; doi:10.1016/j.pccm.2024.05.004)
Supplement: Supplementary file 1 [file mmc1.docx]

**Supplementary Material**

| Supplementary Table 1: Comparison of clinical characteristics of patients with ARDS grouped by VFA/SFA levels before PSM. | | | |
| --- | --- | --- | --- |
| Variables | VFA/SFA <0.73 (*n*=100) | VFA/SFA ≥0.73 (*n*=158) | *P* values |
| Age (years) | 68.00 (48.50, 78.50) | 70.00 (57.00, 78.00) | 0.247 |
| Male | 47 (47.0) | 133 (84.2) | <0.001 |
| BMI (kg/m^2^) | 22.07 (20.28, 26.08) | 23.01 (21.25, 25.71) | 0.134 |
| Smoking history | 22 (22.0) | 49 (31.0) | 0.114 |
| Underlying diseases |  |  |  |
| Hypertension | 50 (50.0) | 95 (60.1) | 0.110 |
| Diabetes | 25 (25.0) | 37 (23.4) | 0.772 |
| Coronary heart disease | 6 (6.0) | 9 (5.7) | 0.919 |
| Hyperlipidemia | 3 (3.0) | 7 (4.4) | 0.803 |
| COPD | 8 (8.0) | 13 (8.2) | 0.948 |
| ARDS classification upon ICU admission | |  | 0.279 |
| Mild | 26 (26.0) | 49 (31.0) |  |
| Moderate | 46 (46.0) | 78 (49.4) |  |
| Severe | 28 (28.0) | 31 (19.6) |  |
| APACHE II score | 14.00 (10.00, 18.00) | 14.50 (11.00, 17.00) | 0.561 |
| SOFA score | 7.00 (5.00, 10.00) | 7.00 (5.00, 10.00) | 0.933 |
| Laboratory indicators upon ICU admission | |  |  |
| Neutrophil count (×10^9^/L) | 9.56 (6.63, 14.56) | 9.20 (5.70, 14.10) | 0.457 |
| Platelet count (×10^9^/L) | 157.50 (99.75, 225.75) | 156.00 (95.00, 226.50) | 0.930 |
| Hemoglobin (g/L) | 113.72±28.60 | 113.84±27.31 | 0.974 |
| Albumin (g/L) | 30.36±5.96 | 29.96±6.37 | 0.623 |
| AST (U/L) | 45.00 (24.00, 95.75) | 36.85 (23.90, 69.25) | 0.154 |
| ALT (U/L) | 27.00 (17.08, 54.50) | 28.50 (17.00, 61.40) | 0.839 |
| Arterial blood gas upon ICU admission | |  |  |
| PaO_2_/FiO_2_ (mmHg) | 168.17 (119.62, 221.59) | 170.00 (112.78, 247.63) | 0.643 |
| Lactate (mmol/L) | 1.75 (1.10, 3.30) | 2.20 (1.40, 3.78) | 0.024 |
| Mechanical ventilation duration (days) | 7.50 (4.00, 15.75) | 9.00 (5.00, 15.00) | 0.305 |
| ICU length of stay (days) | 14.00 (8.00, 25.50) | 14.00 (8.00, 24.00) | 0.838 |
| Outcome |  |  |  |
| In-hospital mortality | 29 (29.0) | 79 (50.0) | 0.001 |
| Data are presented as mean±standard deviation, median (Q_1_, Q_3_) or *n* (%). ALT: Alanine aminotransferase; APACHE: Acute Physiology and Chronic Health Evaluation; ARDS: Acute respiratory distress syndrome; AST: Aspartate aminotransferase; BMI: Body mass index; COPD: Chronic obstructive pulmonary disease; FiO_2_: Fraction of inspired oxygen; ICU: Intensive care unit; MA: Muscle area; PaO_2_: Partial pressure of oxygen in arterial blood; PSM: Propensity score matching; SBW: Standard body weight; SFA: Subcutaneous fat area; SOFA: Sequential Organ Failure Assessment; VFA: Visceral fat area. | | | |

| Supplementary Table 2: Comparison of clinical characteristics of patients with ARDS grouped by MA/SBW levels before PSM. | | | |
| --- | --- | --- | --- |
| Variables | MA/SBW <1.55 cm^2^/kg (*n*=174) | MA/SBW ≥1.55 cm^2^/kg (*n*=84) | *P* values |
| Age (years) | 70.00 (59.00, 79.00) | 65.50 (48.00, 77.50) | 0.023 |
| Male | 121 (69.5) | 59 (70.2) | 0.909 |
| BMI (kg/m^2^) | 21.97 (20.06, 24.22) | 25.30 (22.86, 28.53) | <0.001 |
| Smoking history | 47 (27.0) | 24 (28.6) | 0.793 |
| Previous underlying diseases |  |  |  |
| Hypertension | 98 (56.3) | 47 (56.0) | 0.955 |
| Diabetes | 45 (25.9) | 17 (20.2) | 0.322 |
| Coronary heart disease | 11 (6.3) | 4 (4.8) | 0.780 |
| Hyperlipidemia | 5 (2.9) | 5 (6.0) | 0.230 |
| COPD | 15 (8.6) | 6 (7.1) | 0.684 |
| ARDS classification upon ICU admission |  |  | 0.912 |
| Mild | 52 (29.9) | 23 (27.4) |  |
| Moderate | 83 (47.7) | 41 (48.8) |  |
| Severe | 39 (22.4) | 20 (23.8) |  |
| APACHE II Score | 15.00 (11.00, 18.00) | 13.00 (11.00, 17.00) | 0.048 |
| SOFA Score | 7.00 (5.00, 10.00) | 8.00 (5.00, 10.00) | 0.423 |
| Laboratory indicators upon ICU admission |  |  |  |
| Neutrophil count (×10^9^/L) | 9.25 (5.91, 13.88) | 9.83 (6.19, 14.93) | 0.458 |
| Platelet count (×10^9^/L) | 157.50 (102.00, 227.25) | 156.50 (90.50, 224.75) | 0.715 |
| Hemoglobin (g/L) | 113.72±27.41 | 113.93±28.63 | 0.956 |
| Albumin (g/L) | 29.81±6.17 | 30.76±6.26 | 0.249 |
| AST (U/L) | 38.95 (23.50, 82.95) | 39.50 (24.23, 69.75) | 0.895 |
| ALT (U/L) | 25.65 (15.00, 58.55) | 32.00 (19.70, 62.90) | 0.082 |
| Arterial blood gas upon ICU admission |  |  |  |
| PaO_2_/FiO_2_ (mmHg) | 171.37 (116.01, 250.95) | 168.17 (113.11, 218.83) | 0.398 |
| Lactic acid (mmol/L) | 2.20 (1.40, 3.73) | 1.50 (1.20, 2.48) | 0.010 |
| Duration of mechanical ventilation (days) | 9.00 (5.00, 16.00) | 8.00 (4.25, 14.00) | 0.733 |
| Length of ICU stay (days) | 13.00 (8.00, 23.25) | 14.00 (8.00, 24.75) | 0.400 |
| Outcome |  |  |  |
| In-hospital mortality rate | 93 (53.4) | 15 (17.9) | <0.001 |
| Data are presented as mean±standard deviation, median (Q_1_, Q_3_) or *n* (%). ALT: Alanine aminotransferase; APACHE: Acute Physiology and Chronic Health Evaluation; ARDS: Acute respiratory distress syndrome; AST: Aspartate aminotransferase; BMI: Body mass index; COPD: Chronic obstructive pulmonary disease; FiO_2_: Fraction of inspired oxygen; ICU: Intensive care unit; MA: Muscle area; PaO_2_: Partial pressure of oxygen in arterial blood; PSM: Propensity score matching; SBW: Standard body weight; SFA: Subcutaneous fat area; SOFA: Sequential Organ Failure Assessment; VFA: Visceral fat area. | | | |

| Supplementary Table 3: Comparison of clinical characteristics of patients with pulmonary-origin ARDS grouped by VFA/SFA before PSM. | | | |
| --- | --- | --- | --- |
| Variables | VFA/SFA <1.01 (*n*=102) | VFA/SFA ≥1.01 (*n*=68) | *P* values |
| Age (years) | 71.00 (59.00, 80.00) | 74.00 (69.00, 83.00) | 0.045 |
| Male | 56 (54.9) | 62 (91.2) | <0.001 |
| BMI (kg/m^2^) | 22.80±3.71 | 22.81±3.49 | 0.976 |
| Smoking history | 28 (27.5) | 20 (29.4) | 0.781 |
| Underlying diseases |  |  |  |
| Hypertension | 55 (53.9) | 49 (72.1) | 0.017 |
| Diabetes | 25 (24.5) | 19 (27.9) | 0.617 |
| Coronary heart disease | 9 (8.8) | 4 (5.9) | 0.680 |
| Hyperlipidemia | 2 (2.0) | 1 (1.5) | 1.000 |
| COPD | 11 (10.8) | 7 (10.3) | 0.917 |
| ARDS classification upon ICU admission |  |  | 0.831 |
| Mild | 30 (29.4) | 20 (29.4) |  |
| Moderate | 42 (41.2) | 31 (45.6) |  |
| Severe | 30 (29.4) | 17 (25.0) |  |
| APACHE II score | 14.00 (10.00, 17.00) | 15.00 (12.00, 18.00) | 0.386 |
| SOFA score | 7.00 (5.00, 9.00) | 7.00 (5.00, 10.00) | 0.311 |
| Laboratory indicators upon ICU admission |  |  |  |
| Neutrophil count (×10^9^/L) | 9.67 (8.00, 15.25) | 8.49 (5.58, 14.90) | 0.552 |
| Platelet count (×10^9^/L) | 170.50 (109.75, 241.00) | 151.00 (98.50, 222.25) | 0.170 |
| Hemoglobin (g/L) | 115.37±27.07 | 114.51±24.82 | 0.835 |
| Albumin (g/L) | 30.78±5.69 | 29.86±6.89 | 0.347 |
| AST (U/L) | 36.50 (23.80, 63.78) | 30.20 (20.55, 55.35) | 0.263 |
| ALT (U/L) | 25.50 (16.75, 43.25) | 26.35 (14.93, 61.80) | 0.835 |
| Arterial blood gas upon ICU admission |  |  |  |
| PaO_2_/FiO_2_ (mmHg) | 161.96 (106.89, 215.74) | 164.79 (93.32, 251.65) | 0.952 |
| Lactate (mmol/L) | 2.00 (1.40, 3.30) | 2.15 (1.40, 3.45) | 0.394 |
| Mechanical ventilation duration (days) | 7.00 (4.00, 16.75) | 9.00 (5.25, 16.00) | 0.129 |
| ICU length of stay (days) | 13.00 (6.75, 25.00) | 12.00 (7.25, 22.00) | 0.790 |
| Outcome |  |  |  |
| In-hospital mortality rate | 38 (37.3) | 45 (66.2) | <0.001 |
| Data are presented as mean±standard deviation, median (Q_1_, Q_3_) or *n* (%). ALT: Alanine aminotransferase; APACHE: Acute Physiology and Chronic Health Evaluation; ARDS: Acute respiratory distress syndrome; AST: Aspartate aminotransferase; BMI: Body mass index; COPD: Chronic obstructive pulmonary disease; FiO_2_: Fraction of inspired oxygen; ICU: Intensive care unit; MA: Muscle area; PaO_2_: Partial pressure of oxygen in arterial blood; PSM: Propensity score matching; SBW: Standard body weight; SFA: Subcutaneous fat area; SOFA: Sequential Organ Failure Assessment; VFA: Visceral fat area. | | | |

| Supplementary Table 4: Comparison of clinical characteristics of patients with pulmonary-origin ARDS grouped by MA/SBW before PSM. | | | |
| --- | --- | --- | --- |
| Variables | MA/SBW <1.48 cm^2^/kg (*n*=121) | MA/SBW ≥1.48 cm^2^/kg (*n*=49) | *P* values |
| Age (years) | 72.00 (63.00, 81.00) | 76.00 (65.50, 82.00) | 0.328 |
| Male | 84 (69.4) | 34 (69.4) | 0.997 |
| BMI (kg/m^2^) | 21.99±3.44 | 24.81±3.25 | <0.001 |
| Smoking history | 32 (26.4) | 16 (32.7) | 0.415 |
| Underlying diseases |  |  |  |
| Hypertension | 68 (56.2) | 36 (73.5) | 0.036 |
| Diabetes | 30 (24.8) | 14 (28.6) | 0.610 |
| Coronary heart disease | 7 (5.8) | 6 (12.2) | 0.151 |
| Hyperlipidemia | 1 (0.8) | 2 (4.1) | 0.200 |
| COPD | 12 (9.9) | 6 (12.2) | 0.655 |
| ARDS classification upon ICU admission |  |  | 0.786 |
| Mild | 37 (30.6) | 13 (26.5) |  |
| Moderate | 50 (41.3) | 23 (46.9) |  |
| Severe | 34 (28.1) | 13 (26.5) |  |
| APACHE II score | 14.00 (11.00, 18.00) | 15.00 (10.00, 17.00) | 0.606 |
| SOFA score | 7.00 (5.00, 9.00) | 7.00 (5.00, 10.00) | 0.420 |
| Laboratory indicators upon ICU admission |  |  |  |
| Neutrophil count (×10^9^/L) | 9.52 (5.95, 13.98) | 9.20 (5.20, 13.71) | 0.597 |
| Platelet count (×10^9^/L) | 165.00 (113.50, 236.50) | 157.00 (93.00, 225.00) | 0.521 |
| Hemoglobin (g/L) | 114.57±25.82 | 116.16±27.10 | 0.720 |
| Albumin (g/L) | 29.99±6.02 | 31.45±6.55 | 0.166 |
| AST (U/L) | 36.00 (23.00, 67.70) | 31.00 (20.50, 54.00) | 0.238 |
| ALT (U/L) | 25.00 (15.40, 54.50) | 27.90 (15.00, 52.50) | 0.989 |
| Arterial blood gas upon ICU admission |  |  |  |
| PaO_2_/FiO_2_ (mmHg) | 166.21 (103.78, 251.30) | 155.15 (97.05, 214.07) | 0.192 |
| Lactate (mmol/L) | 2.20 (1.40, 3.55) | 1.50 (1.20, 2.70) | 0.020 |
| Mechanical ventilation duration (days) | 9.00 (5.00, 16.00) | 8.00 (5.00, 16.50) | 0.663 |
| ICU length of stay (days) | 12.00 (7.00, 23.00) | 14.00 (7.00, 23.50) | 0.376 |
| Outcome |  |  |  |
| In-hospital mortality rate | 74 (61.2) | 9 (18.4) | <0.001 |
| Data are presented as mean±standard deviation, median (Q_1_, Q_3_) or *n* (%). ALT: Alanine aminotransferase; APACHE: Acute Physiology and Chronic Health Evaluation; ARDS: Acute respiratory distress syndrome; AST: Aspartate aminotransferase; BMI: Body mass index; COPD: Chronic obstructive pulmonary disease; FiO_2_: Fraction of inspired oxygen; ICU: Intensive care unit; MA: Muscle area; PaO_2_: Partial pressure of oxygen in arterial blood; PSM: Propensity score matching; SBW: Standard body weight; SFA: Subcutaneous fat area; SOFA: Sequential Organ Failure Assessment; VFA: Visceral fat area. | | | |
